# Supplementary material for: Wireless neuromodulation in vitro and in vivo by intrinsic TRPC-mediated magnetomechanical stimulation
Source: Commun Biol. 2022 Nov 2;5:1166. doi: 10.1038/s42003-022-04124-y (PMC9630493; doi:10.1038/s42003-022-04124-y)
Supplement: Supplementary file 2 — Supplementary Information [file 42003_2022_4124_MOESM2_ESM.pdf]

Supplementary Information for

Wireless neuromodulation *in vitro* and *in vivo* by intrinsic TRPC-mediated magnetomechanical stimulation

Chih-Lun Su[1,2], Chao-Chun Cheng[1,2], Ping-Hsiang Yen[1], Jun-Xuan Huang[1], Yen-Jing Ting[1], Po-Han Chiang[1]*

[1]Institute of Biomedical Engineering, National Yang Ming Chiao Tung University, Hsinchu City, Taiwan (R.O.C.)

[2]These authors contributed equally
*corresponding author, Email: phc@nycu.edu.tw

This PDF file includes:

Supplementary Figure S1 to S8
Supplementary Table S1 to S3
Supplementary Methods

Supplementary Figures

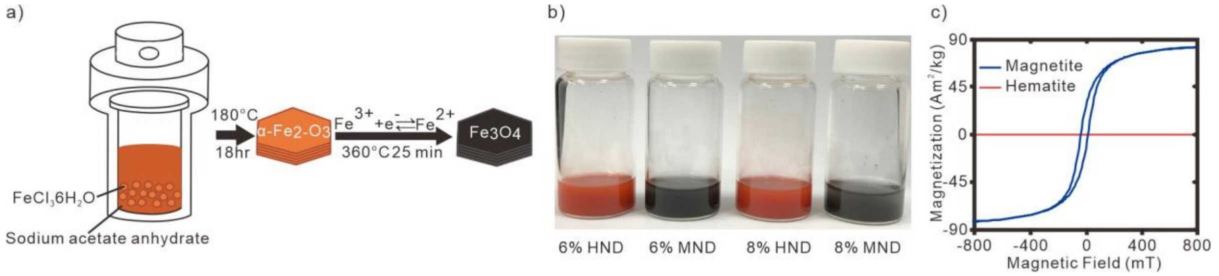

Figure S1, Preparation of magnetic nanodiscs.
a) Schematic of the two steps synthesis process of magnetic nanodiscs. **b)** A photo of HNDs and MNDs with different H_2O concentration in the solution of the first reaction. **c)** Magnetization curves for HNDs (red) and MNDs (blue) by VSM.

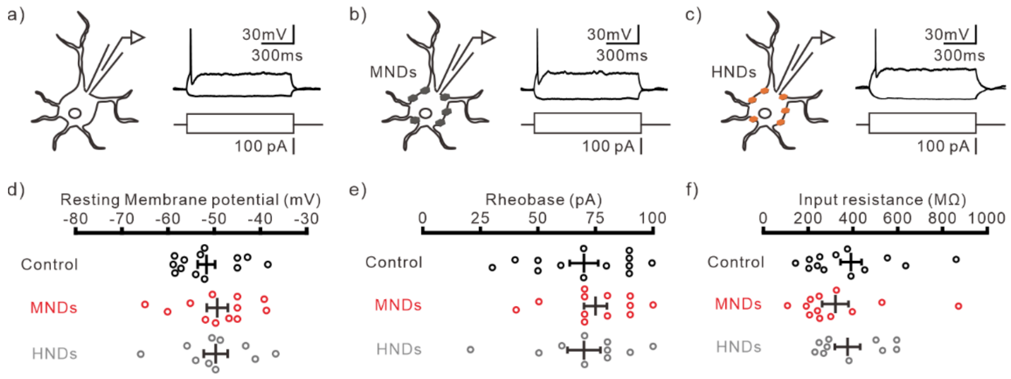

Figure S2, Electrophysiology properties of cultured neurons with MNDs or HNDs.
a) to **c)** Left, schematic of whole-cell recording from cultured neurons without nanodiscs **(a)**, with MNDs **(b)** and with HNDs **(c)**. Top-right, the representative traces of rheobase and input resistance recording. Bottom-right, current steps for the rheobase and input resistance recording at the top-right. **d)** Summary of resting membrane potentials from cultured neurons with or without nanodiscs (Control, n = 13; MNDs, n = 12; HNDs, n = 10). F = 1.397, p = 0.497, Kruskal-Wallis test). **e)** Summary of rheobases from cultured neurons with or without nanodiscs (Control, n = 13; MNDs, n = 12; HNDs, n = 10; F = 0.263, p = 0.877, Kruskal-Wallis test). **f)** Summary of input resistances from cultured neurons with or without nanodiscs (Control, n = 13; MNDs, n = 12; HNDs, n = 10). F = 1.962, p = 0.375, Kruskal-Wallis test). Error bars represent mean ± s.e.m.

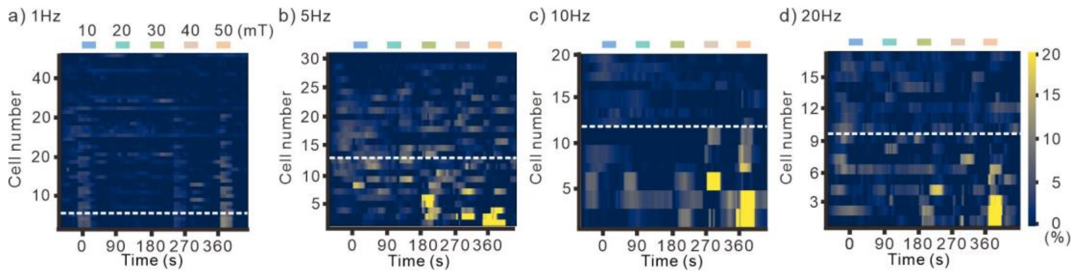

Figure S3, The neuronal activity with different magnetic parameters.

a) to **d)** The heat maps of the Ca^{2+} responses in individual neurons with magnetomechanical stimulation by sequentially increased alternative magnetic fields from 10 to 50 mT at 1 Hz (**a**, n = 46/ 6 (neurons/ samples)), 5 Hz (**b**, n = 30/ 6), 10 Hz (**c**, n = 19/ 6), and 20 Hz (**d**, n = 18/ 6).

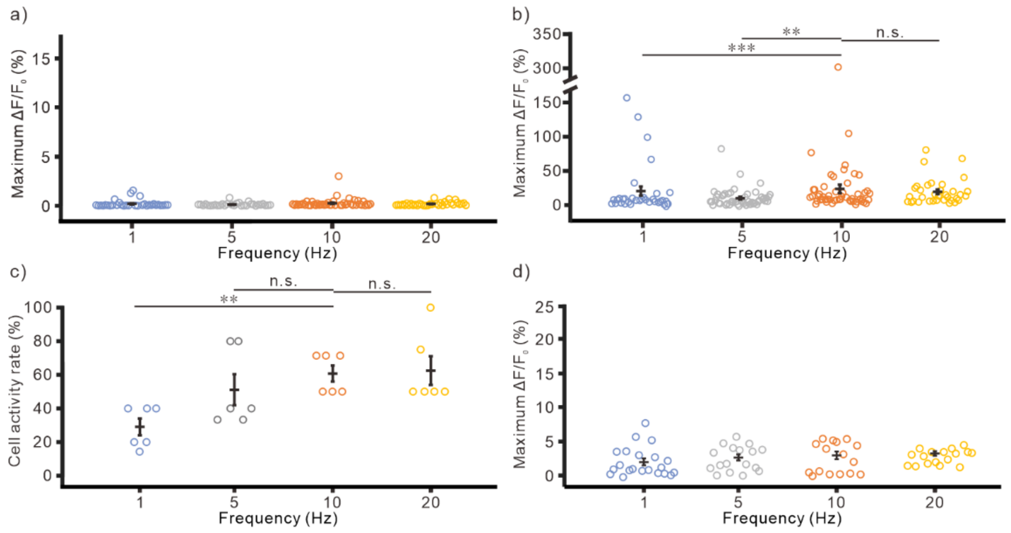

Figure S4, The neuronal activity with different frequency parameters.

a) Maximum $\Delta F/F_0$ of HND treated neurons with single magnetic stimulation at different frequencies (1 Hz, n = 32/ 6 (neurons/ samples); 5 Hz, n = 31/ 6; 10 Hz, n = 44/ 6; 20 Hz, n = 33/ 6). $F = 0.999$, $p = 0.268$, Kruskal-Wallis test. **b)** Maximum $\Delta F/F_0$ of MNDs treated neurons with 4 magnetic stimulations at different frequencies (1 Hz, n = 46/ 6 (neurons/samples); 5 Hz, n = 30/ 6; 10 Hz, n = 19/ 6; 20 Hz, n = 18/ 6). $F = 1.05$, $p = 0.003$, Kruskal-Wallis test. $**p < 0.01$, $***p < 0.001$, Dunn post-hoc test. **c)** Cell activity rate of MNDs treated neurons with 4 magnetic stimulations at different frequencies (All groups, n = 6). $F = 8.208$, $p = 0.005$, Kruskal-Wallis test. $***p < 0.001$, Dunn post-hoc test. **d)** Maximum $\Delta F/F_0$ of HND treated neurons with 4 magnetic stimulations at different frequencies (1Hz, n = 32/ 6 (neurons/ samples); 5Hz, n = 31/ 6; 10Hz, n = 44/ 6; 20Hz, n = 33/ 6). $F = 0.597$, $p = 0.392$, Kruskal-Wallis test. Error bars represent mean ± s.e.m.

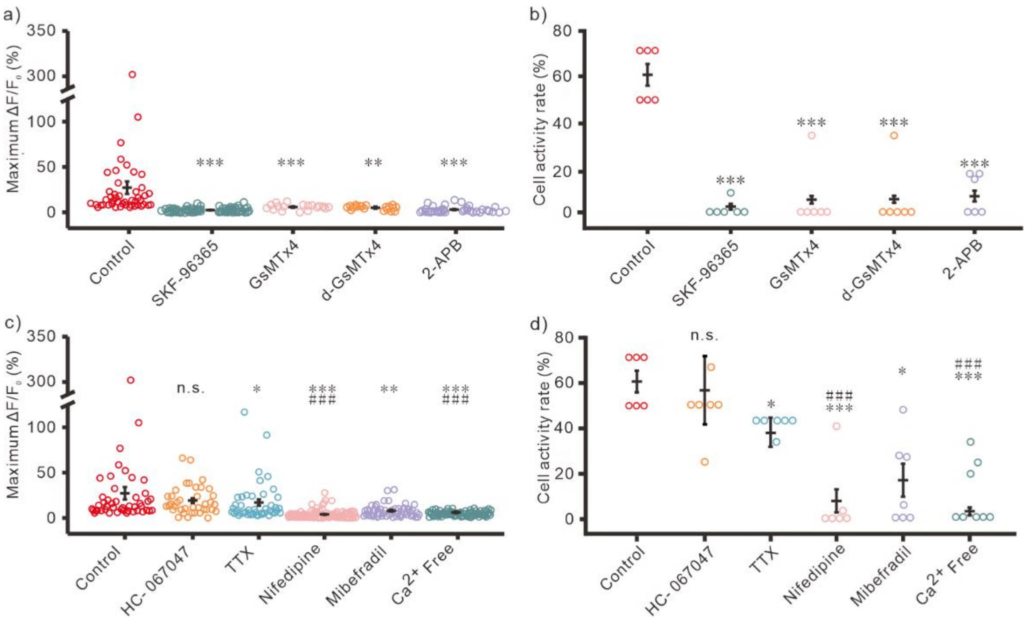

Figure S5, Pharmacological dissection of magnetomechanical stimulated response.

a) Maximum $\Delta F/F_0$ with four magnetic stimulations of MND treated neurons with different TRPC blockers (Control, n = 52/ 6 (neurons/ samples); SKF-96365, n = 73/ 6; GsMTx4, n = 31/ 6; d-GsMTx4, n = 28/ 6; 2-APB, n = 37/ 6). F = 184.483, $p < 0.001$, Kruskal-Wallis test. **b)** Cell activity rate with four magnetic stimulations of MND treated neurons with different TRPC blockers (All groups, n = 6). F = 32.821, $p < 0.001$, Kruskal-Wallis test. **c)** Maximum $\Delta F/F_0$ with four magnetic stimulations of MND treated neurons with different solutions application (Control, n = 52/ 6 (neurons/ samples); HC-067047, n = 38/ 6; TTX, n = 44/ 6; nifedipine, n = 107/ 6; mibefradil, n = 50/ 7; Ca^{2+}-free solution, n = 79/ 8). F = 135.911, $p < 0.001$, Kruskal-Wallis test. **d)** Cell activity rate with four magnetic stimulations of MND treated neurons with different solutions application (Control, n = 6; HC-067047, n = 6; TTX, n = 6; nifedipine, n = 6; mibefradil, n = 7; Ca^{2+}-free solution, n = 8)). F = 30.674, $p < 0.001$, Kruskal-Wallis test. *$p < 0.05$, **$p < 0.01$, ***$p < 0.001$, compared to the control group; ##$p < 0.01$, ###$p < 0.001$, compared to HC-67047 group; Dunn post-hoc test. Error bars represent mean ± s.e.m.

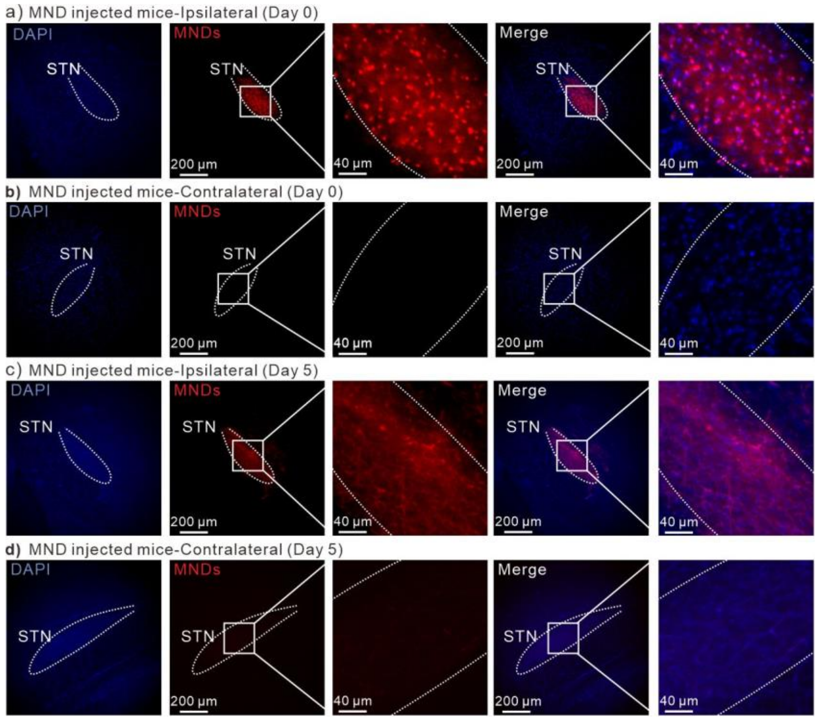

Figure S6, The coordinate of Alexa 594-conjugated MNDs in STN of mice.
a) to **d)** The image from left to right is DAPI, fluorescence-labeled MNDs, enlarged image of fluorescence-labeled MNDs, merged image and enlargement of merged image in STN of fluorescence-labeled MNDs injected mice. The brain slices were collected at the same day of (**a-b**) or at 5 days after injection (**c-d**).

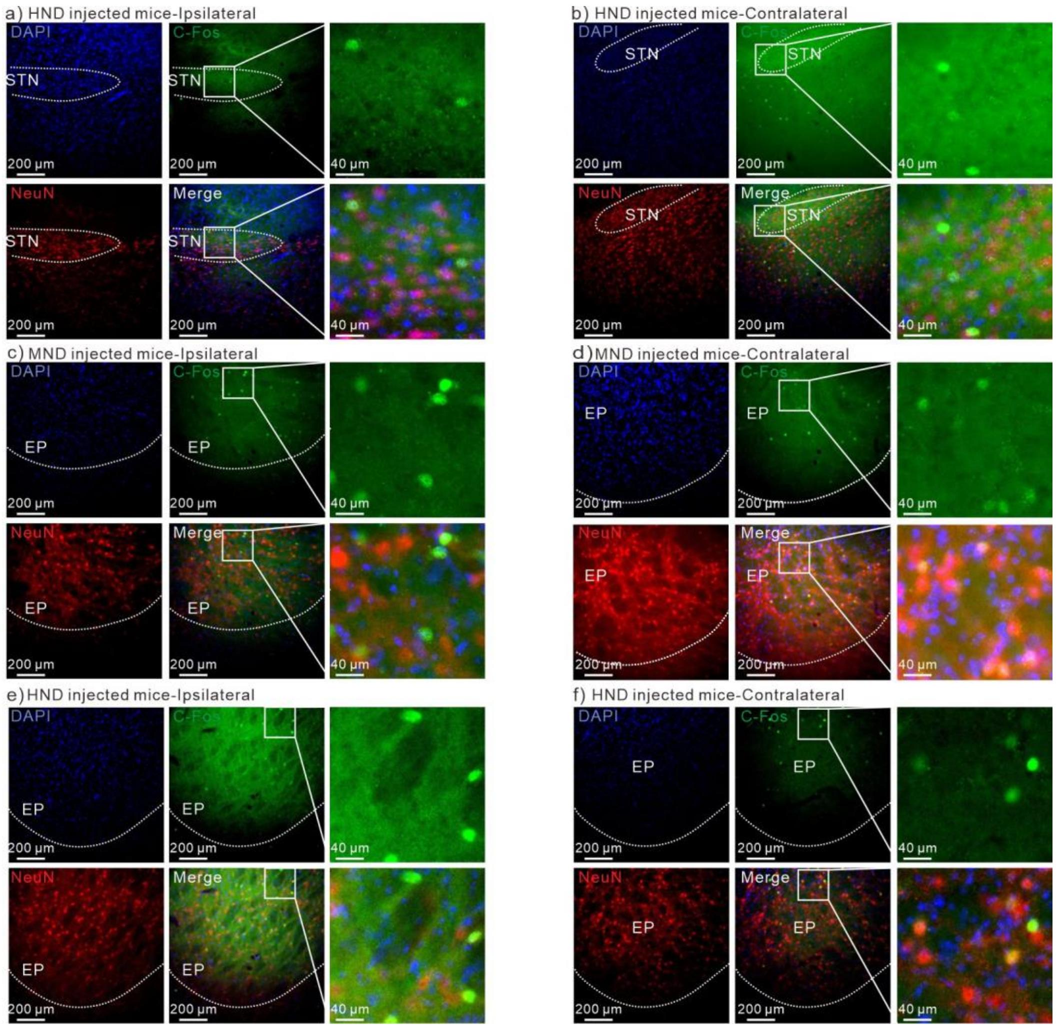

Figure S7, c-fos expression in STN and EP of MND or HND injected mice.

a) to **f**) Immunostaining of DAPI (top-left), c-fos (top-middle), enlarged image of c-fos (top-right), NeuN (bottom-left), merged image (bottom-middle) and enlarged image of merged image (bottom-right) in STN of HND injected mice (**a-b**), EP of MND injected mice (**c-d**), and EP of HND injected mice (**e-f**) after magnetic stimulations.

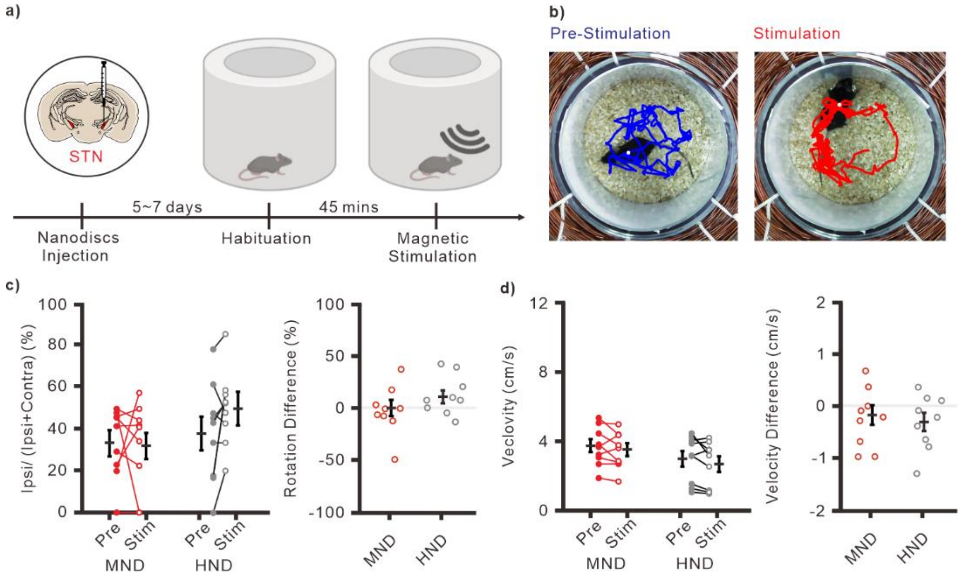

Figure S8, Rotation behavior of mice with MNDs and HNDs injected at STN.

a) Schematic of behavior experiment. **b**) The position of the mice's body during the behavior experiment. 30-second interval trajectories of mice movements before magnetic stimulation (Pre-Stimulation, left) and during magnetic stimulation (Stimulation, right). **c**) Ratio of ipsilateral rotations to total number of rotations before and during magnetic stimulation (left, $p = 0.132$ for MNDs, $p = 0.724$ for HNDs, Wilcoxon signed-rank test), and difference between them (right, $p = 0.834$, Mann-Whitney U test) in MND and HND injected mice first time stimulation on 7 days after injection in STN (MND injected mice, $n = 17$; HND injected mice, $n = 14$). **d**) Velocity before and during magnetic stimulation (left, $p = 0.854$ for MNDs, $p = 0.296$ for HNDs, Wilcoxon signed-rank test), and difference between them (right, $p = 0.279$, Mann-Whitney U test) in MND and HND injected mice first time stimulation on 7 days after injection in STN (MND injected mice, $n = 17$; HND injected mice, $n = 14$). Error bars represent mean ± s.e.m.

Supplementary Tables

	6 % H_2O	8 % H_2O
w/ PMAO	p = 0.173/ p = 0.280	p = 0.940/ p = 0.734
w/o PMAO	p = 0.762/ p = 0.353	p = 0.427/ p = 0.029

Table S1, Statistical analysis of diameter or thickness between HNDs and MNDs.
Mann-Whitney test was used for statistical analysis of diameter and thickness between HNDs and MNDs (p value of diameter/ p value of thickness). The summary dot plot is demonstrated in figure 1f-g. n = 10 in each group.

	6 % H_2O	8 % H_2O
Magnetite	p = 0.739/ p = 0.796	p = 0.290/ p = 0.218
Hematite	p = 0.733/ p = 0.739	p = 0.161/ p = 0.003

Table S2, Statistical analysis of diameter or thickness between PMAO-coated and PMAO-uncoated nanodiscs.
Mann-Whitney test was used for statistical analysis of diameter between PMAO-coated and PMAO-uncoated nanodiscs (p value of diameter/ p value of thickness). The summary dot plot is demonstrated in figure 1f-g. n = 10 in each group.

	w/ PMAO	w/o PMAO
Magnetite	p < 0.001/ p < 0.001	p < 0.001/ p < 0.001
Hematite	p < 0.001/ p < 0.001	p = 0.001/ p < 0.001

Table S3, Statistical analysis of diameter or thickness between nanodiscs from 6 % H_2O and 8 % H_2O in solution of first step synthesis.
Mann-Whitney test was used for statistical analysis of diameter between nanodiscs from 6 % H_2O and 8 % H_2O in solution of first step synthesis (p value of diameter/ p value of thickness). The summary dot plot is demonstrated in figure 1f-g. n = 10 in each group.

Supplementary Materials and Methods

Arduino script for controlling in vivo coil system

Arduino Uno board was used to control the alternative magnetic field for the *in vivo* coil system. The code was written by Arduino IDE (version 1.8.13) and was compiled for Arduino Uno and Arduino Mega. In this program, phases of two signals for 4 full-bridge drivers had 180° difference. The pins 4 to 7 of Arduino Uno were used for one signal to generate the same orientation of magnetic fields in 4 coils. The pins 8 to 11 of Arduino Uno were used for the other signal to generate the reversed magnetic fields in 4 coils. Serial BAUD rate set at 500000 for stimulation trigger and monitor on computer.

```
void setup() {
  Serial.begin(500000);
  pinMode(4,OUTPUT);//control1
  pinMode(5,OUTPUT);//control2
  pinMode(6,OUTPUT);//control3
  pinMode(7,OUTPUT);//control4
  pinMode(8,OUTPUT);//control1
  pinMode(9,OUTPUT);//control2
  pinMode(10,OUTPUT);//control3
  pinMode(11,OUTPUT);//control4
  pinMode(13,OUTPUT);//indicate light
}
void loop() {
  // put your main code here, to run repeatedly:
  allStop();
  readFromPC();
}
void freqstart(){
  digitalWrite(4,HIGH);//control1
  digitalWrite(5,HIGH);//control2
  digitalWrite(6,HIGH);//control3
  digitalWrite(7,HIGH);//control4
  digitalWrite(8,LOW);//control1
  digitalWrite(9,LOW);//control2
  digitalWrite(10,LOW);//control3
  digitalWrite(11,LOW);//control4
  digitalWrite(13,HIGH);//test
  delay(100);//100->5Hz; 50 -> 10hz; 25 -> 20hz
  digitalWrite(4,LOW);//control1
  digitalWrite(5,LOW);//control2
  digitalWrite(6,LOW);//control3
  digitalWrite(7,LOW);//control4
  digitalWrite(8,HIGH);//control1
  digitalWrite(9,HIGH);//control2
  digitalWrite(10,HIGH);//control3
  digitalWrite(11,HIGH);//control4
```

```
  digitalWrite(13,LOW);//test
  delay(100);//100->5Hz; 50 -> 10hz; 25 -> 20hz
}
 void allStop(){ // Both IN1 & IN2 LOW
  digitalWrite(8,LOW);//IN2
  digitalWrite(9,LOW);//IN2
  digitalWrite(10,LOW);//IN2
  digitalWrite(11,LOW);//IN2
  digitalWrite(4,LOW);//IN1
  digitalWrite(5,LOW);//IN1
  digitalWrite(6,LOW);//IN1
  digitalWrite(7,LOW);//IN1
}
void readFromPC(){
    String s = "";
    int i = 0;
    int j = 0;
    int k = 0;
    while (Serial.available()) {
        char c = Serial.read();
        if(c!=""){
        Serial.println(c);
        }
        if(c!='\n'){
          if(c == 'a'){
            Serial.println("start stimulate");
            for(j=1;j<=5;j++){
              Serial.print("doing ");
              Serial.println(j);
              for(i=0;i<150;i++){   //5hz=> i=150; 10hz=> 300 for 30s;
                freqstart();
              }
               Serial.print("done");
               Serial.println(j);
               allStop();
               delay(60000);
            }
            Serial.println("alldone");
          }
        }
        //delayMicroseconds(2);    //change BUAD to 500000, no need delay anymore
    }
}
```

Python script for fluorescence image measurement

After the calcium images were captured from a fluorescence microscope, the videos were processed with a custom python script based on opencv2 and numpy. This custom python script consists of a user interface which is based on wxpython and cvui for adjusting the parameters for measurements. The cells are autoselected by the following process: “Blur”, “AdaptiveThreshold”, “Erosion”, “Dilation” and “findContours”. The mean value of intensity in individual cells are measured and output as a “.txt” file for further analysis.

```
__author__ = 'Po-Han Chiang, NYCU'
__version__ = '0.19'
__status__ = 'Developing'

import pickle
import copy
import cv2, cvui, imutils #for showing and calculate images
import numpy as np #for arrays
import os, fnmatch #for checking file exist
import wx, wx.lib.intctrl
import threading
from collections import deque
from multiprocessing.pool import ThreadPool
import time
#for ignore some warnings=====================================
import warnings
warnings.simplefilter(action='ignore', category=DeprecationWarning)
warnings.simplefilter(action='ignore', category=np.VisibleDeprecationWarning)



class VideoReader():
    def __init__(self,parent):
        self.mainPanel = parent
        self.resetParameters()
        self.runVideo = False


    def saveParameters(self, parameters): #[blurSize, m_blocksize, open_iter, dilate_iter,
th_blocksize, th_meanc, distance_ratio, sizelimit]
        pkl_filename = 'CaImage reader Parameters.pkl'
        with open(pkl_filename, 'wb') as file: #write file
            pickle.dump(parameters, file)

    def loadParameters(self, save_defult = True):
        pkl_filename = 'CaImage reader Parameters.pkl'
        try:
            with open(pkl_filename, 'rb') as file: #read file
                pkl_parameters = pickle.load(file)
```

```
            if isinstance(pkl_parameters, list) and len(pkl_parameters)==2:
                return pkl_parameters
            else:
                return copy.deepcopy(self.parameters_defult)
        except:
            return copy.deepcopy(self.parameters_defult)

    def cellMeasurementInit(self,input_filename):
        self.filename = input_filename
        _, filebasename = os.path.split(self.filename)
        self.resetParameters()#reset parameters
        self.saveData = SaveFunc() #for save data
        self.runVideo = True

        cap = cv2.VideoCapture(self.filename)#read the video
        _, img_test = cap.read()

        if img_test is not None:
            self.mainPanel.updateText('Start Analyzer:  %s\n'%filebasename)
            self.cellMeasurement(cap)
            self.mainPanel.updateText('End Analyzer:  %s\n'%filebasename)

        else:
            self.mainPanel.updateText('ERROR! File Damaged:  %s\n'%filebasename)

    def cellMeasurement(self,videocapture):
        cap = videocapture
        _, filebasename = os.path.split(self.filename)
        window_name = filebasename ###cvui
        cvui.init(window_name, 20)
        self.v_time = [0] #reset the time for video
        self.contours_original = [] #reset
        self.contours == [] #reset
        self.contours_unwanted = [] #reset




        self.total_frame_no = cap.get(cv2.CAP_PROP_FRAME_COUNT)#total frame numbers in
the image
        #read the first frame=====================================
        cap.set(cv2.CAP_PROP_POS_FRAMES,self.v_time[0])
        _, img_o = cap.read()
        img = img_o.copy()
        img_gray = cv2.cvtColor(img.astype('uint8'),cv2.COLOR_BGR2GRAY)
```

```
        img_color = np.repeat(np.expand_dims(img_gray.copy(),axis = -1),3,axis = -1) #expand to
3 channels
        #--------------------------------------------------------

        thread_num = cv2.getNumberOfCPUs()#how many cores
        if thread_num > 1:
            self.multicore = True

        pool = ThreadPool(processes=thread_num)#define threadpools for process image
        pending_task = deque()

        while self.runVideo:

            #reading img from each time, avoid error in the end of
video=====================
            try:
                cap.set(cv2.CAP_PROP_POS_FRAMES,self.v_time[0])#reset frame number for
reading
                _,img_o = cap.read()
                img = img_o.copy()

            except:#at when self.v_time larger than the videos
                self.pause = True

            #------------------------------------------------------------------------------
##              if cap.get(cv2.CAP_PROP_POS_FRAMES) == v_time - 1

            img_gray = cv2.cvtColor(img.astype('uint8'),cv2.COLOR_BGR2GRAY)


            if self.multicore and not self.pause:
                task = pool.apply_async(self.imageProcess, (img_gray.copy(),self.v_time[0],))
                pending_task.append(task)

                while len(pending_task) > 0 and pending_task[0].ready():
                    img_show = pending_task.popleft().get()
                    cv2.imshow(window_name,img_show)
            else:
                img_show = self.imageProcess (img_gray.copy(),self.v_time[0])
                cv2.imshow(window_name,img_show)
            #######
            ##-------------------------------------------------------------------------------------------

            if not self.pause:
                self.v_time[0] += 1
```

```
            k = cv2.waitKey(1) & 0xFF

            if k ==27: #close with esc button
                break
            elif k == 32: #space for pausing videos
                self.pause = not self.pause
            elif k == 112: #p for printing informations
                printing = not printing
            elif k == 115: #s for saving file
                output_filename = self.saveData.setFilename(self.filename,'_brightness_','.txt')
                self.saveData.save2File(self.brightness_all,output_filename)
            elif k <255:
                print(k)

            if not cv2.getWindowProperty(window_name, cv2.WND_PROP_VISIBLE): #close
video with "x" button
                print('close window')
                break



        cap.release()
        cv2.destroyAllWindows()
        self.runVideo = False

    def disableAllFunction(self):
        self.finding_cell = False
        self.finding_deleteCell = False
        self.recover_deleteCell = False
        self.draw_separatecell = False
        self.getting_brightness = False
    def appendNumpy(self,array1,array2): #append a new cell to original contours numpy array
        if array1 == [] or array1.shape[0] == 0:
            return np.array([array2])
        else:
            output = []
            for i in range(array1.shape[0]):
                output.append(array1[i])
                if i == array1.shape[0] - 1:
                    output.append(array2)
            return np.array(output)
    def dist2AllContours(self,contours,point):#find distance of all contour to the mouse point
        dist_all = np.array([])
        for cnt in contours:
            dist = cv2.pointPolygonTest(cnt,tuple(point),True)
            dist_all = np.append(dist_all, dist)
```

```
        dist_all[dist_all<0] = 9999
        return dist_all
    def detectTransform(prev_gray,curr_gray):

        # Detect feature points in previous frame
        prev_pts = cv2.goodFeaturesToTrack(prev_gray, maxCorners=200, qualityLevel=0.01
,minDistance=30 ,blockSize=3) #goodFeaturesToTrack
        # Calculate optical flow (i.e. track feature points)
        curr_pts, status, err = cv2.calcOpticalFlowPyrLK(prev_gray, curr_gray, prev_pts, None)
#calcOpticalFlowPyrLK
        # Sanity check
        assert prev_pts.shape == curr_pts.shape
        # Filter only valid points
        idx = np.where(status==1)[0]
        prev_pts = prev_pts[idx]
        curr_pts = curr_pts[idx]
        #Find transformation matrix
        m,_ = cv2.estimateAffinePartial2D (prev_pts, curr_pts) #will only work with OpenCV-3 or
less #estimateRigidTransform
        # Extract traslation
        dx = m[0,2]
        dy = m[1,2]
        # Extract rotation angle
        da = np.arctan2(m[1,0], m[0,0])
        return [dx,dy,da]



    def imageProcess(self,img_gray, time_point):
        img_color = np.repeat(np.expand_dims(img_gray.copy(),axis = -1),3,axis = -1) #expand to
3 channels
        if self.finding_cell:#finding outline of cells
            #stop other functions====
            self.disableAllFunction()#disalbe all other function
            self.finding_cell = True
            self.contours_original = []
            self.contours == [] #reset
            self.contours_unwanted = [] #reset
            #------------------------
            self.contours = self.findCell(img_gray,self.findCell_parameters)
            self.contours_original = self.contours.copy()

        if self.finding_deleteCell and not self.contours == []:##choose unwanted cells
            self.disableAllFunction()#disalbe all other function
            self.finding_deleteCell = True

            if cvui.mouse().x > self.left_boarder:#when mouse in the image
```

```
            point = np.array([cvui.mouse().x - self.left_boarder,
cvui.mouse().y])*img_color.shape[1]/self.display_parameters[0] #resize the point to the related
point in image
            point = point.astype(int)

            dist2cnts = self.dist2AllContours(self.contours,point)

            if not np.sum(dist2cnts) == 9999*len(dist2cnts):#find the contour with mouse inside
                target_cell = self.contours[np.argmin(dist2cnts)]
                cv2.drawContours(img_color,[np.squeeze(target_cell)],-1,(150,150,255),-1)#show
the contour with mouse inside
                if cvui.mouse(cvui.UP): #save delete cell after click
                    self.contours_unwanted = self.appendNumpy(self.contours_unwanted ,
target_cell)
                    self.contours = np.delete(self.contours,np.argmin(dist2cnts),0)#remove unwanted
cells

        if not self.contours_unwanted == []:#show unwanted cells
            for cnt in self.contours_unwanted:
                cv2.drawContours(img_color, [cnt], 0, (200,200,255), -1)

    if self.recover_deleteCell and not self.contours_unwanted == []:##recover deleted cells
        self.disableAllFunction()#disalbe all other function
        self.recover_deleteCell = True

        for cnt in self.contours_unwanted:#show unwanted cells first
            cv2.drawContours(img_color, [cnt], 0, (200,200,255), -1)

        if cvui.mouse().x > self.left_boarder:#when mouse in the image

            point = np.array([cvui.mouse().x - self.left_boarder,
cvui.mouse().y])*img_color.shape[1]/self.display_parameters[0] #resize the point to the related
point in image
            point = point.astype(int)

            dist2cnts = self.dist2AllContours(self.contours_unwanted,point)
            if not np.sum(dist2cnts) == 9999*len(dist2cnts):#find the contour with mouse inside
                target_cell = self.contours_unwanted[np.argmin(dist2cnts)]
                cv2.drawContours(img_color,[np.squeeze(target_cell)],-1,(150,255,255),-1)#show
the contour with mouse inside
                if cvui.mouse(cvui.UP): #save delete cell after click
                    self.contours = self.appendNumpy(self.contours, target_cell)#add cell back
                    self.contours_unwanted =
np.delete(self.contours_unwanted,np.argmin(dist2cnts),0)#remove unwanted cells
```

```
        if self.draw_separatecell and not self.contours == []:##choose unwanted cells
            self.disableAllFunction()#disalbe all other function
            self.draw_separatecell = True
            if cvui.mouse().x > self.left_boarder:#when mouse in the image
                point = np.array([cvui.mouse().x - self.left_boarder,
cvui.mouse().y])*img_color.shape[1]/self.display_parameters[0] #resize the point to the related
point in image
                point = point.astype(int)

                if cvui.mouse(cvui.DOWN):
                    self.temp_line[0] = point
                    self.mouse_wasdown = True
                elif cvui.mouse(cvui.IS_DOWN) and self.mouse_wasdown:
                    self.temp_line[1] = point

cv2.line(img_color,tuple(self.temp_line[0]),tuple(self.temp_line[1]),(100,255,255),3)
                elif cvui.mouse(cvui.UP) and self.mouse_wasdown:

                    self.draw_lines.append(self.temp_line)
                    self.temp_line = [[0,0],[0,0]]
                    self.mouse_wasdown = False

            if self.draw_lines != []:
                cnt_mask = np.zeros(img_gray.shape,'uint8')
                for cnt in self.contours_temp:
                    cv2.drawContours(cnt_mask, [cnt], 0, (255), -1) #draw cells in the mask image
                    cv2.drawContours(cnt_mask, [cnt], 0, (0), 2) #draw cells in the mask image
                for line in self.draw_lines:
                    cv2.line(cnt_mask,tuple(line[0]),tuple(line[1]),(0), self.line_thickness[0]) #draw
black lines in mask image
                    cv2.line(img_color,tuple(line[0]),tuple(line[1]),(100,255,255),
self.line_thickness[0]) #draw lines in original image

                foundcnts, _ =
cv2.findContours(cnt_mask,cv2.RETR_TREE,cv2.CHAIN_APPROX_SIMPLE)
                foundcnts = [cnt for cnt in foundcnts if cv2.contourArea(cnt) >
self.findCell_parameters[7]]
                self.contours = foundcnts


        if self.getting_brightness and not self.contours == []:#measure brightness of cells
            self.disableAllFunction()#disalbe all other function
            self.getting_brightness = True

            brightness = np.zeros(len(self.contours)) #
            time_tag = ['time:'+str(time_point)]#set the first value as frame number
```

```
            for i, cnt in enumerate(self.contours):

                x, y, w, h = cv2.boundingRect(cnt) #find a rectangle around the contour
                img_patch = img_gray[y:y+h,x:x+w] #extract the patch of image from
                cnt_mask = np.zeros(img_patch.shape,'uint8') #create a mask with same size
                cv2.drawContours(cnt_mask, [cnt-[x,y]], 0, (255), -1) #draw cell on mask
                brightness[i] = cv2.mean(img_patch,cnt_mask)[0] #calculate the average brightness of
cell

            brightness_tag = np.concatenate((time_tag,brightness))
            ##connect all data==================================================
            if np.ndim(self.brightness_all) == 1:
                self.brightness_all.append(brightness_tag)
            else:
                self.brightness_all = [brightness_tag if x[0]==brightness_tag[0] else x for x in
self.brightness_all]
                if time_tag not in np.array(self.brightness_all)[:,0]:
                    self.brightness_all.append(brightness_tag)
            ##------------------------------------------------------------------


        if not self.hide_image:
            img_show = cv2.convertScaleAbs(img_color.copy(), alpha = self.display_parameters[1],
beta = self.display_parameters[2]) #change contrast
            if self.contours != [] and not self.hide_contours:
                for i, cnt in enumerate(self.contours):

                    self.display_parameters[3] = self.display_parameters[3] if
self.display_parameters[3] > 0 else 1
                    cv2.drawContours(img_show, [cnt], 0, (0,0,255), self.display_parameters[3]) #draw
cell in the original image
                    cnt_center = np.mean(np.squeeze(cnt),axis = 0)
                    cv2.putText(img_show, str(i+1),tuple(cnt_center.astype('int16')),
cv2.FONT_HERSHEY_SIMPLEX, self.display_parameters[4], (0, 0,255),
self.display_parameters[3], 255)#add cell number to image

            img_show = imutils.resize(img_show,self.display_parameters[0])#resize image
            img_show = cv2.copyMakeBorder(img_show, 0, 0, self.left_boarder, 0,
cv2.BORDER_CONSTANT, None, (0,0,0))##add boarder for widgets(source, top, bottom, left,
right, boardertype, none, boardercolor)
        else:
            img_show = np.zeros((750, 750, 3), np.uint8)
```

```
        ##add cvui widgets to
image================================================================
================
        if cvui.button(img_show, 10, 10, 50, 30, 'Play' if self.pause else 'Pause'):
            self.pause = not self.pause


        cvui.trackbar(img_show, 60, 0, 440, self.v_time, 0, self.total_frame_no, 0, '%d',
self.trackbar_options, 1)
        if cvui.button(img_show, 10, 50, 120, 30, 'Adjust'):
            if not self.adjust_image:
                self.pause = True
                self.modify_cell = False
                self.adjust_image = True ##
                self.finding_cell = False
                self.getting_brightness = False
            else:
                self.adjust_image = False

        if cvui.button(img_show, 130, 50, 120, 30, 'Find'):
            if not self.finding_cell:
                self.pause = True
                self.modify_cell = False
                self.adjust_image = False
                self.finding_cell = True ##for find cells
                self.getting_brightness = False
                self.findCell([],destroy_windows = True)
            else:
                self.finding_cell = False


        if cvui.button(img_show, 250, 50, 120, 30, 'Modify'):
            if not self.modify_cell:
                self.pause = True
                self.adjust_image = False
                self.finding_cell = False
                self.getting_brightness = False
                self.modify_cell = True ##
                self.finding_deleteCell = False
                self.draw_separatecell = False
            else:
                self.modify_cell = False
                self.finding_deleteCell = False
                self.draw_separatecell = False
        if self.modify_cell:
            if cvui.button(img_show, 10, 100, 120, 30, 'Delete Cell'):
```

```
        self.finding_deleteCell = not self.finding_deleteCell
        self.recover_deleteCell = False
        self.draw_separatecell = False
    if self.finding_deleteCell:
        if cvui.button(img_show, 10, 130, 120, 30, 'Undo'):
            if not self.contours_unwanted == [] and not self.contours_unwanted.shape[0] == 0:
                cnt = self.contours_unwanted[-1]
                self.contours_unwanted = self.contours_unwanted[:-1]
                self.contours = self.appendNumpy(self.contours, cnt)#add cell back
        if cvui.button(img_show, 10, 160, 120, 30, 'Reset'):
            if not self.contours_unwanted == [] and not self.contours_unwanted.shape[0] == 0:
                for cnt in self.contours_unwanted:
                    self.contours = self.appendNumpy(self.contours, cnt)#add cell back
                self.contours_unwanted = []
        if not self.contours == []:##choose unwanted cell
            cvui.printf(img_show, 10, 200, 0.5, 0xFFFFFF, 'Click the cell to delete')
        else:
            cvui.printf(img_show, 10, 200, 0.5, 0xFFFFFF, 'Please find cells first')

    if cvui.button(img_show, 130, 100, 120, 30, 'Recover Cell'):
        self.recover_deleteCell = not self.recover_deleteCell
        self.finding_deleteCell = False
        self.draw_separatecell = False

    if self.recover_deleteCell:
        if not self.contours_unwanted == []:##choose unwanted cell
            cvui.printf(img_show, 10, 200, 0.5, 0xFFFFFF, 'Click the deleted cells to recover')
        elif self.contours == [] and self.contours_unwanted == []:
            cvui.printf(img_show, 10, 200, 0.5, 0xFFFFFF, 'Please find cells first')
        elif not self.contours == [] and self.contours_unwanted == []:
            cvui.printf(img_show, 10, 200, 0.5, 0xFFFFFF, 'Please delete cells first')


    if cvui.button(img_show, 370, 100, 120, 30, 'Separate Cell'):
        self.draw_separatecell = not self.draw_separatecell
        self.finding_deleteCell = False
        self.contours_temp = self.contours
    if self.draw_separatecell:
        cvui.printf(img_show, 10, 200, 0.5, 0xFFFFFF, 'Draw a line to separate cells')
        cvui.text(img_show, 10, 230, 'Line Thickness = %d' % self.line_thickness[0])
        cvui.counter(img_show, 200, 230, self.line_thickness, 1, '%d')
        if self.line_thickness[0] < 1:
            self.line_thickness[0] = 1

        if cvui.button(img_show, 370, 130, 120, 30, 'Undo'):
            self.draw_lines = self.draw_lines[:-1]
```

```
                if self.draw_lines == []:
                    self.contours = self.contours_temp
            if cvui.button(img_show, 370, 160, 120, 30, 'Reset'):
                self.draw_lines = []
                self.contours = self.contours_temp
        else:
            self.draw_separatecell = False
            self.finding_deleteCell = False




        if cvui.button(img_show, 370, 50, 120, 30, 'Measure'):

            self.modify_cell = False
            self.finding_cell = False
            self.adjust_image = False
            self.findCell([],destroy_windows = True)
            if not self.getting_brightness:
                self.getting_brightness = True
                self.v_time[0] = 0 #reset video
                self.brightness_all = [] #reset data
                self.pause = False
            else:
                self.getting_brightness = False
                self.pause = True

        if self.getting_brightness:
            cvui.printf(img_show, 10, 90, 'Play the video to measure the brightness')
            output_filename = self.saveData.setFilename(self.filename,'_brightness_','.txt')
            if cvui.button(img_show, 10, 150, 100, 30, 'Save'):
                cvui.printf(img_show, 10, 120, output_filename)
                self.saveData.save2File(self.brightness_all,output_filename)


        if self.adjust_image:
            show_value = [self.display_parameters[0]]
            cvui.printf(img_show, 10, 90, 'Resize Image Width = %d' % show_value[0])
            cvui.trackbar(img_show, 10, 100, 350, show_value, 256, 2048, 0, '%d',
self.trackbar_options, 1)
            cvui.counter(img_show, 400, 110, show_value, 1, '%d')
            self.display_parameters[0] = show_value[0]


            show_value = [self.display_parameters[1]]
            cvui.printf(img_show, 10, 150, 'Alpha = %.2f (Defult = 1)' % show_value[0])
```

```
            cvui.trackbar(img_show, 10, 160, 350, show_value, 1, 5, 0,
'%.2f',cvui.TRACKBAR_HIDE_SEGMENT_LABELS |
cvui.TRACKBAR_HIDE_STEP_SCALE)
            cvui.counter(img_show, 400, 170, show_value, 0.01, '%.2f')
            self.display_parameters[1] = show_value[0]

            show_value = [self.display_parameters[2]]
            cvui.printf(img_show, 10, 210, 'Beta = %d (Defult = 0)' % show_value[0])
            cvui.trackbar(img_show, 10, 220, 350, show_value, -200, 200, 0,
'%.2f',self.trackbar_options,1)
            cvui.counter(img_show, 400, 230, show_value, 1, '%d')
            self.display_parameters[2] = show_value[0]

        if self.finding_cell:
            findCell_parameters_lowlimit = [1,1,1,1,1,-255]
            findCell_parameters_uplimit = [51,51,51,51,2048,255]
            findCell_parameters_label = ['Image Blur Block Size = %d',
                                         'Morphological Kernel Size = %d',
                                         'Opening Iteration Size = %d',
                                         'Dilation Iteration Size = %d',
                                         'Adaptive Threshold Blocksize = %d',
                                         'Adaptive Threshold Mean + C  = %d']

            for i in range(6):
                widget_y = 90 + (i * 60)
                show_value = [self.findCell_parameters[i]]
                cvui.printf(img_show, 10, widget_y, findCell_parameters_label[i] % show_value[0])
                cvui.trackbar(img_show, 10, widget_y + 10, 350, show_value,
findCell_parameters_lowlimit[i], findCell_parameters_uplimit[i], 0, '%d', self.trackbar_options,
2)
                if (show_value[0] % 2) == 0: show_value[0] +=1
                cvui.counter(img_show, 400, widget_y + 20, show_value, 2, '%d')
                self.findCell_parameters[i] = show_value[0]

            widget_y = 90 + (6 * 60)
            show_value = [self.findCell_parameters[6]]
            cvui.text(img_show, 10, widget_y, 'Distance Ratio = %.1f' % show_value[0])
            cvui.trackbar(img_show, 10, widget_y+10, 350, show_value, -1, 1, 0,
'%.1f',self.trackbar_options, 0.1)
            cvui.counter(img_show, 400, widget_y+20, show_value, 0.1, '%.1f')
            self.findCell_parameters[6] = show_value[0]

            widget_y = 90 + (7 * 60)
            show_value = [self.findCell_parameters[7]]
            cvui.text(img_show, 10, widget_y, 'Cell Size Limit = %d' % show_value[0])
```

```
            cvui.trackbar(img_show, 10, widget_y+10, 350, show_value, 0, 2048, 0, '%d',
self.trackbar_options, 1)
            cvui.counter(img_show, 400, widget_y+20, show_value, 1, '%d')
            self.findCell_parameters[7] = show_value[0]

            show_value = [self.display_parameters[3]]
            cvui.text(img_show, 10, 115+480, 'Contour Thickness = %d' % show_value[0])
            cvui.counter(img_show, 180, 110+480, show_value, 1, '%d')
            self.display_parameters[3] = show_value[0]

            show_value = [self.display_parameters[4]]
            cvui.text(img_show, 290, 115+480, 'Font Size = %d' % show_value[0])
            cvui.counter(img_show, 400, 110+480, show_value, 1, '%d')
            self.display_parameters[4] = show_value[0]

        frameRows,frameCols,frameChannels = img_show.shape

        if cvui.button(img_show, 10, frameRows - 80, 120, 30, 'TurnOff Multicore' if self.multicore
else 'TurnOn Multicore'):
            self.multicore = not self.multicore
        if cvui.button(img_show, 130, frameRows - 80, 120, 30, 'Hide Image' if not self.hide_image
else 'Show Image'):
            self.hide_image = not self.hide_image
        if cvui.button(img_show, 250, frameRows - 80, 120, 30, 'Hide Contours' if not
self.hide_contours else 'Show Contours'):
            self.hide_contours = not self.hide_contours
        if cvui.button(img_show, 370, frameRows - 80, 120, 30, 'Save Image'):#save image with
self.contours
            imagename = self.saveData.setFilename(self.filename,'_contours_','.tiff')
            img_save = cv2.convertScaleAbs(img_color.copy(), alpha = self.display_parameters[1],
beta = self.display_parameters[2]) #change contrast
            if self.contours != [] and not self.hide_contours:
                for i, cnt in enumerate(self.contours):
                    cv2.drawContours(img_save, [cnt], 0, (0,0,255), self.display_parameters[3]) #draw
cell in the original image
                    cnt_center = np.mean(np.squeeze(cnt),axis = 0)
                    cv2.putText(img_save, str(i+1),tuple(cnt_center.astype('int16')),
cv2.FONT_HERSHEY_SIMPLEX, self.display_parameters[4], (0, 0,255),
self.display_parameters[3], 255)#add cell number to image

            self.saveImage(imagename, img_save)


        if cvui.button(img_show, 10, frameRows - 40, 120, 30, '&Quit'):
            self.runVideo = False
        if cvui.button(img_show, 130, frameRows - 40, 120, 30, 'Defult'):
```

```
            [self.findCell_parameters, self.display_parameters] =
copy.deepcopy(self.parameters_defult)

        if cvui.button(img_show, 250, frameRows - 40, 120, 30, 'Load Parameters'):#save
findcell_parameters
            [self.findCell_parameters, self.display_parameters] = self.loadParameters()

        if cvui.button(img_show, 370, frameRows - 40, 120, 30, 'Save Parameters'):#save
findcell_parameters
            self.saveParameters([self.findCell_parameters, self.display_parameters])

        cvui.update()
        return img_show

    def resetParameters(self):
        #parameters for choose unwanted cells====
        self.finding_deleteCell = False
        self.recover_deleteCell = False
        self.save_deleteCell = False
        self.contours_unwanted = []
        self.draw_separatecell = False
        self.line_thickness = [10] #line thickness for separate cells
        self.temp_line = [[0,0],[0,0]]#lines for separate cells
        self.draw_lines = []#lines for separate cells
        self.mouse_wasdown = False
        #----------------------------------------
        #parameters for finding cells ===========
        self.parameters_defult = [[9, 5, 1, 3, 501, -9, 0.1, 100], [750, 1.0, 0, 3, 3]]
        #[[blurSize, m_blocksize, open_iter, dilate_iter, th_blocksize, th_meanc, distance_ratio,
sizelimit],
        # [resize_pixel, contrast_alpha, contrast_beta, contour_thickness, font_size]]
        [self.findCell_parameters, self.display_parameters] =
copy.deepcopy(self.parameters_defult)
        self.findCell_parameters = [9, 5, 1, 3, 501, -9, 0.1, 100]
        self.display_parameters = [750, 1.0, 0, 3, 3]
        self.pause = True #for pausing video
        self.hide_contours = False
        self.adjust_image = False
        self.finding_cell = False #for find cells
        self.getting_brightness = False #for measuring brightness of cell
        self.modify_cell = False
        #parameters for display=======
        self.hide_image = False
        #for analysis========================
        self.contours = [] #outline for cells
        self.contours_temp = self.contours
```

```
##      self.brightness = [] #averaged self.brightness for each cells
        self.brightness_all = [] #averaged self.brightness for All cells
        self.left_boarder = 500
        self.trackbar_options = cvui.TRACKBAR_DISCRETE |
cvui.TRACKBAR_HIDE_SEGMENT_LABELS | cvui.TRACKBAR_HIDE_STEP_SCALE
        self.v_time = [0] #set the time for video
        self.total_frame_no = 1
        self.saveData = SaveFunc() #for save data

    def saveImage(self, filename, image):
        cv2.imwrite(filename, image)
    def findCell(self,img_input, input_parameters = [9, 5, 1, 3, 61, -9, 0.2, 30], destroy_windows
= False):
        [blurSize, m_blocksize, open_iter, dilate_iter, th_blocksize, th_meanc, distance_ratio,
sizelimit] = input_parameters
        if destroy_windows:
            cv2.destroyWindow('threshold image')
            cv2.destroyWindow('cell_full')
        else:
            img_blur = cv2.blur(img_input,(blurSize,blurSize))###need to check how much to
blur###
            img_th =
cv2.adaptiveThreshold(img_blur,255,cv2.ADAPTIVE_THRESH_MEAN_C,cv2.THRESH_BIN
ARY,th_blocksize,th_meanc)#threshold the img
            img_show = img_th.astype('uint8')
####        cv2.imshow('threshold image',img_th)
            ##===get the center of cells=======
            kernel = np.ones((m_blocksize,m_blocksize),np.uint8)
            opening = cv2.morphologyEx(img_th,cv2.MORPH_OPEN,kernel, iterations = open_iter)
            opening = opening.astype('uint8')
            sure_bg = cv2.dilate(opening,kernel,iterations = dilate_iter)  #background
            dist_transform = cv2.distanceTransform(opening,cv2.DIST_L2,5) #find distance to edge
for each point
            _, sure_fg = cv2.threshold(dist_transform,distance_ratio*dist_transform.max(),255,0)
#threshold the distance to edge #########
            sure_fg = np.uint8(sure_fg) #foreground
            ##--------------------------------
            unknown = cv2.subtract(sure_bg,sure_fg)
            # Marker labelling
            ## find the number, markers, sizes, and centers of cells
==========================
            count, markers,sizes,centers = cv2.connectedComponentsWithStats(sure_fg)
            count -= 1 #delete the outside box
            sizes=sizes[1:,-1]
            centers=centers[1:,:].astype(int)
            ##-------------------------------------------------------------------------------
```

```
##		img_show = np.copy(img_input)
		markers = markers+1
		markers[unknown==255] = 0
		img_forws = np.repeat(np.expand_dims(img_input.copy(), axis = -1), 3, axis = -1)#make
correct dimension for watershed

		markers = cv2.watershed(img_forws,markers)
		cell_full = np.ones(img_input.shape,'uint8')*255
		cell_full[markers >1] = 0 #
		#-----------------------------------------------------------------------------------------
		cell_count = 0

####print(cv2.findContours(cell_full,cv2.RETR_TREE,cv2.CHAIN_APPROX_NONE))#####
		foundcnts, _ =
cv2.findContours(cell_full,cv2.RETR_TREE,cv2.CHAIN_APPROX_SIMPLE)
		foundcnts = [cnt for cnt in foundcnts if cv2.contourArea(cnt) > sizelimit]

		foundcnts = foundcnts[1:]#remove the frame edge

		return foundcnts
class SaveFunc():
    def __init__(self):
        pass
    def save2File(self,data_array, savefilename):
        newfile = open(savefilename, 'w')
        for line in data_array:
            newline = self.editLine(str(line))
            newfile.write(str(newline))
        newfile.close()
    def editLine(self,line):####need to check when copy to other scripts
        newline = line
        newline = newline.replace('[','')
        newline = newline.replace('\'time:','')
        newline = newline.replace(' ','')
        newline = newline.replace('\n','')
        newline = newline.replace('\'\'','\t')
        newline = newline.replace('\']','\n')
        return newline
    def setFilename(self,fullname,addname, extname):
        filedir, filebasename = os.path.split(fullname)
        filebasename, filetype = filebasename.split('.')
        newname = ''
        filenumber = 0
        while newname == '':
            filenumberstr = str(filenumber)
            for i in range(4-len(filenumberstr)): filenumberstr = '0'+ filenumberstr
```

```
        tempname =  filedir+'\\'+filebasename + addname +filenumberstr + extname
        if os.path.isfile(tempname):
            filenumber = filenumber + 1
        else:
            newname = tempname
    return newname
##==========================================================================
==============================================



class MyFileDropTarget(wx.FileDropTarget):

    def __init__(self, parent):

        wx.FileDropTarget.__init__(self)
        self.mainPanel = parent

    def OnDropFiles(self, x, y, filenames):
        self.mainPanel.updateText("File Name:\n")
        self.mainPanel.updateText(filenames[0] + '\n')
        self.mainPanel.targetname = filenames[0]


        return True #??? why need to return a bool??? ######


class MainPanel(wx.Panel): #panel for DnD

    def __init__(self, parent):
        wx.Panel.__init__(self, parent=parent) #initialize
        self.parent = parent
        file_drop_target = MyFileDropTarget(self) #call function MyFileDropTarget
        self.SetDropTarget(file_drop_target)
        self.targetname = ''
        self.vr = VideoReader(self)
        self.createWidget()
    def disableAllButton(self):
        self.anaButton.Disable()
    def enableAllButton(self):
        self.anaButton.Enable()
    def createWidget(self):

        self.fileTextCtrl = wx.TextCtrl(self, size = (300, 200),
                                style=wx.TE_MULTILINE|wx.HSCROLL|wx.TE_READONLY)#add
text region for drop file
```

```
        self.fileTextCtrl.WriteText('Drag a calcium image video to here. \n')

        self.anaButton = wx.Button(self, size = (300, 25),label="Analyzer")
        self.anaButton.Bind(wx.EVT_BUTTON, self.anaButton_press)

        self.mainSizer = wx.BoxSizer(wx.VERTICAL) #difine sizer

        self.mainSizer.Add(self.fileTextCtrl, 0,wx.LEFT|wx.EXPAND,2) #add widget
        self.mainSizer.Add(self.anaButton, 0, wx.LEFT|wx.EXPAND, 2) #add widget
        self.SetSizer(self.mainSizer) #set


    def anaButton_press(self,event):
        filetypes = ['tif','avi','mp4']
        if self.targetname.split('.')[-1] in filetypes:
            if not self.vr.runVideo:
                time.sleep(1)
                t = threading.Thread

                t.__target = self.vr.cellMeasurementInit(self.targetname)
                try:
                    t.start()
                except:
                    if not self.vr.runVideo:
                        pass
                    else:
                        self.vr.runVideo = False
                #----------------------------------------------------
            else:
                self.fileTextCtrl.WriteText('ERROR! Close another video before start \n')
        else:
            self.fileTextCtrl.WriteText('NOTE! Please drag and drop a video \n')


    def updateText(self, text):
        self.fileTextCtrl.SetInsertionPointEnd()
        self.fileTextCtrl.WriteText(text)#Write text to the text control




#----------------------------------------------------------------------
#main frame for UI=====================================================
class MainFrame(wx.Frame):
    def __init__(self):
```

```
        wx.Frame.__init__(self, parent=None, title="CaImageReader",size=(500,265))
        panel = MainPanel(self) #panel for DnD
        self.Show()

#----------------------------------------------------------------------
if __name__ == "__main__":
    app = wx.App(False)
    frame = MainFrame()
    app.MainLoop()
```

Fluorescence intensity analysis

After the fluorescence intensity of individual cells are measured from a custom python script. Another custom python script based on numpy was used for converting fluorescence intensity into $\Delta F/F_0$. Time dependent baseline F0 where used for calculating the change of fluorescence intensity. First, smoothed fluorescence intensity F(t) by averaging of raw data from 5 s period for each time point.

$$F(t) = \frac{1}{\tau_0}\int_{t-\frac{\tau_0}{2}}^{t+\frac{\tau_0}{2}} F(\tau)d\tau \quad \text{, where } \tau_0 = 5 \text{ s} \tag{1}$$

The minimum value of smoothed F(t) during 30 s time period before each time point t was taken as the time dependent basline F0(t).

$$F_0(t) = \{min(F(t))|((t-\tau_1) < x < t)\} \text{, where } \tau_1 = 30 \text{ s} \tag{2}$$

The relative change of fluorescence intensity was calculated from F(t) and F0(t)

$$R(t) = \frac{F(t)-F_0(t)}{F_0(t)} \tag{3}$$

In final $\Delta F/F_0(t)$, the noise was filtered by exponentially weighted moving average with 3 s time period.

$$\frac{\Delta F}{F_0}(t) = \frac{\int_0^t R(t-\tau)\cdot w(\tau)d\tau}{\int_0^t w(\tau)d\tau} \tag{4}$$

Where,

$$w(\tau) = e^{-\frac{\tau}{\tau_2}} \text{, where } \tau_2 = 30 \text{ s} \tag{5}$$

The activated cell was defined by the cell with maximum ΔF/F0 more than 10 % at indicated time periods.

```
__author__ = 'Po-Han Chiang'
import wx
```

```
import matplotlib.pyplot as plt
import numpy as np
from pandas import Series as pdS #for emwa filter
import os
from threading import Thread
import webbrowser


class TracePlot():
    def __init__(self,parent):
        self.sf = saveFunc()
        self.parent = parent
    def dataPlotShow(self,filename,parameters = [10,30,3,180,180],
                     actcheck = [False,True,True,False],
                     pltcheck = [True,True,True,True],
                     heatmap_style = 'rainbow'):
        #parameters is number of [average length, search length, exponential weighted moving
average, baseline length, stimulation start point]
        #pltcheck is bool of [raw data,all traces,averaged trace, heatmap,active cell numbers]
        #==name each input===========
        self.filename = filename
        filebasename = os.path.basename(self.filename).split('.')[0]

        mean_len = parameters[0]
        search_len= parameters[1]
        EWMA_num = parameters[2]
        baseline_len = parameters[3]
        stim_start = parameters[4]
        removecell_check = actcheck[0]
        bwd_sub_check = actcheck[1]
        savedFF0_check = actcheck[2]
        saveAve_check = actcheck[3]

        #----------------------------

        traces = self.dataRead(self.filename)

##          print(np.shape(traces))
        if stim_start > baseline_len:
            remove_len = stim_start - baseline_len
            traces = traces[:,remove_len-1:]
        elif stim_start < baseline_len:
            baseline_len = stim_start

        tm = TraceModify()
```

```
        all_data_bwd_sub =
tm.EWMAfilter(tm.TraceBgSub(traces,bwd_sub_check,mean_len,search_len),EWMA_num)
##          self.dataPlot('raw dF/F0:',all_data_bwd_sub)

        all_std = self.stdAllPoints(all_data_bwd_sub[1:,:baseline_len])

        if pltcheck[0]: self.dataPlot(filebasename,'Raw data',traces) #plot raw data
        #==remove shifted cells====================
        if removecell_check:
            all_traces = self.removeShiftedCells(all_data_bwd_sub,all_std,5) #remove sudden
dropped traces
        else:
            all_traces = all_data_bwd_sub



        if pltcheck[1]: self.dataPlot(filebasename,'dF/F0',all_traces)

        #==averaged trace==========================
        all_traces_ave = self.dataAve(all_traces.copy())
        if savedFF0_check:
            self.sf.save2File(np.transpose(all_traces),self.sf.setFilename(filename,'_dFF0_','.txt'))
#save to file
        if saveAve_check:

self.sf.save2File(np.transpose(all_traces_ave),self.sf.setFilename(filename,'_dFF0_Ave_','.txt'))
#save to file
        if pltcheck[2]: self.dataPlot(filebasename,'Averaged trace',all_traces_ave)
        if pltcheck[3]: self.heatmapPlot(filebasename,heatmap_style, all_traces)#plot heat map for
all traces


        #==counting activated cell numbers=========

        plt.show()

    def removeShiftedCells(self,data,all_std,stdtimes = 5):
        all_traces = data[1:].copy()
        all_traces = np.array([trace for i, trace in enumerate(all_traces) if np.min(trace)> -
all_std*stdtimes])
        all_traces = np.vstack((data[0],all_traces)) #put frame number back
        return all_traces
    def stdAllPoints(self,data):
        #==get std from all points===============
        all_traces = data.copy()
        all_points = all_traces.flatten()
```

```
        return np.std(all_points)
        #--------------------------------------
    def activeCells(self,data,frame_stim = 116):
        print(data.shape)
        frame_num = data[0]
        #get std from each cells
        frame_start = int(frame_num[0])
        baseline_len = frame_stim - frame_start + 1
        baseline_all = data[1:,:baseline_len]
        ave_ind = np.mean(baseline_all,axis = 0)
        std_ind = np.std(baseline_all,axis = 0)

        print(ave_ind.shape, std_ind.shape)
##        sem_ind =



    def dataAve(self,data):
        frame_num = data[0]
        traces = data[1:]
        ave_trace = np.mean(traces,axis = 0)
        output = np.vstack((frame_num,ave_trace))
        return output
    def dataRead(self,filename):
        all_data = []
        for line in open(filename,'r'):
            values = [float(s) for s in line.split()]
            all_data.append(values)
        all_data = np.transpose(np.array(all_data))#transpose data, row 0 will be frame number
        return all_data

    def dataPlot(self,title, pltname, data ):
        plt.style.use('fast') #change style of plot
        plt.rcParams["font.family"] = "arial" #change font of plot
        plt.rcParams["font.size"] = 16 #change font size of plot
        plt.rcParams["figure.figsize"] = (4,3) #change size of plot
        '''
        style of plot can check this website:
        https://matplotlib.org/stable/gallery/style_sheets/style_sheets_reference.html
        '''
        plt.figure(title+'_'+ pltname)
        plt.title(title+'_'+ pltname)
        for i in range(len(data)-1):
            x = data[0]
            y = data[i+1]
            plt.plot(x,y)
```

```
        plt.plot(x,y)


    def heatmapPlot(self,title,heatmapstyle, data):
        plt.style.use('fast') #change style of plot
        plt.rcParams["font.family"] = "arial" #change font of plot
        plt.rcParams["font.size"] = 16 #change font size of plot
        plt.rcParams["figure.figsize"] = (4,3) #change size of plot

        plt.figure(title +'_heatmap')
        plt.title(title +'_heatmap')
        x_mesh = data[0] #seperate frame number from data
        heat = data[1:]
        y_mesh = range(1,len(heat)+1)#name each cells
        try:
            plt.pcolormesh(x_mesh, y_mesh, heat, shading='nearest',
                           cmap=heatmapstyle, #the color code for heatmap
                           vmin=heat.min(), vmax=heat.max()) #automatic scale: vmin=heat.min(),
vmax=heat.max()
        except:
            self.parent.heatmap_style_Txt.SetValue('rainbow')
            plt.pcolormesh(x_mesh, y_mesh, heat, shading='nearest',
                           cmap='rainbow', #the color code for heatmap
                           vmin=heat.min(), vmax=heat.max()) #automatic scale: vmin=heat.min(),
vmax=heat.max()

        '''
        style of heatmap (cmap) can check this website:
        https://matplotlib.org/stable/gallery/color/colormap_reference.html
        '''
        plt.colorbar()




    def stop(self):
        self._is_running = False

##class activeCellCount(data):
##    def __init__(self,data):
##        pass


class TraceModify():
    def __init__(self):
        pass
```

```
    def TraceBg(self,data,backward = True, mean_len = 10,search_len = 30):
        if backward: bwd = 1
        else: bwd = -1
        data_len = len(data[0])
        data_mean = self.aveTraces(data,mean_len)##average of each point with indicated length
        ##find min of mean======================
        if backward:
            data_mean = np.array(data_mean[:,data_len-1::-1])
            data_mean_min = np.transpose([np.min(data_mean[:,i:i+search_len], axis = -1) for i in
range(len(data_mean[0]))])[:,::-1]
        else:
            data_mean = np.array(data_mean[:,mean_len::])
            data_mean_min = np.transpose([np.min(data_mean[:,i:i+search_len], axis = -1) for i in
range(len(data_mean[0]))])
        data_mean_min[0] = data[0] #recover frame numbers
        return data_mean_min

    def TraceStd(self,data,backward = True, mean_len = 10,search_len = 30):
        if backward: bwd = 1
        else: bwd = -1
        data_len = len(data[0])
        data_std = self.stdTraces(data,mean_len)
        if backward:
            data_std = np.array(data_std[:,data_len-1::-1])
        else:
            data_std = np.array(data_std[:,mean_len::])
        data_std [0] = data[0]
        return data_std

    def stdTraces(self,data,mean_len = 10):
        data_len = len(data[0])
        ##average of each point with indicated length================================
        data_std_begin = np.transpose(np.array([np.std(data[:,0:i+1], axis = -1) for i in
range(mean_len)])) #average of the begining of traces
        data_std_center = np.transpose(np.array([np.std(data[:,i:i+mean_len], axis = -1) for i in
range(len(data[0]) - mean_len)])) #average of the begining of traces
        data_std_end = np.transpose(np.array([np.std(data[:,data_len-i-1:data_len], axis = -1)for i in
range(mean_len)]))[:,::-1]  #average of the begining of traces
        output = np.append(np.append(data_std_begin,data_std_center,axis = -1),data_std_end,axis
= -1)
        return output
    def aveTraces(self,data,mean_len = 10):
        data_len = len(data[0])
        ##average of each point with indicated length================================
        data_mean_begin = np.transpose(np.array([np.mean(data[:,0:i+1], axis = -1) for i in
range(mean_len)])) #average of the begining of traces
```

```
        data_mean_center = np.transpose(np.array([np.mean(data[:,i:i+mean_len], axis = -1) for i in
range(len(data[0]) - mean_len)])) #average of the begining of traces
        data_mean_end = np.transpose(np.array([np.mean(data[:,data_len-i-1:data_len], axis = -
1)for i in range(mean_len)]))[:,::-1]  #average of the begining of traces
        output = np.append(np.append(data_mean_begin,data_mean_center,axis = -
1),data_mean_end,axis = -1)
        return output
    def dFF0(self,data,background):
        data_bg_sub = np.true_divide(np.subtract(data, background), background)
        data_bg_sub[0] = data[0]#recover the frame number
        return data_bg_sub

    def TraceBgSub(self,data,backward = True, mean_len = 10,search_len = 30):
        all_data_bg_sub = self.dFF0(data,self.TraceBg(data,backward,mean_len,search_len))
        return all_data_bg_sub

    def EWMAfilter(self,data, filter_range = 3):
        output_data = data
        for i in range(len(data)-1):
            trace = data[i+1]
            df = pdS(trace) #transform to pandas readable form
            fwd = pdS.ewm(df,span=filter_range).mean() #ewma filter the forward trace
            bwd = pdS.ewm(df[::-1],span=filter_range).mean() #ewma filter the backward trace
            output_data[i+1] = np.mean(np.vstack(( fwd, bwd[::-1] )), axis=0 ) #average fwd and
bwd
        return output_data

class saveFunc():
    def __init__(self):
        pass
    def save2File(self,data_array, savefilename):
        newfile = open(savefilename, 'w')
        for line in data_array:
            newline = self.editLine(str(line))
            newfile.write(str(newline))
        newfile.close()
    def editLine(self,line):####need to check when copy to other scripts
        newline = line
        newline = ' '.join(newline.split(' '))
        newline = newline.replace('['or '[ ','')
        newline = newline.replace('\n ',' ')
        newline = ' '.join(newline.split())
        newline = newline.replace(' ','\t')
        newline = newline.replace(']','\n')

        return newline
```

```
    def setFilename(self,fullname,addname, extension):
        filedir, filebasename = os.path.split(fullname)
        filename = filebasename.split('.')
        filetype = filename[-1]
        filebasename = filebasename[:-len(filetype)-1]
        newname = ''
        filenumber = 0
        while newname == '':
            filenumberstr = str(filenumber)
            for i in range(4-len(filenumberstr)): filenumberstr = '0'+ filenumberstr
            tempname =  filedir+'\\'+filebasename + addname +filenumberstr + extension
            if os.path.isfile(tempname):
                filenumber = filenumber + 1
            else:
                newname = tempname
        return newname
class MyFileDropTarget(wx.FileDropTarget):
    """"""
    #----------------------------------------------------------------------
    def __init__(self, window):
        wx.FileDropTarget.__init__(self)
        self.window = window

    #----------------------------------------------------------------------
    def OnDropFiles(self, x, y, filenames):
        """
        When files are dropped, write where they were dropped and then
        the file paths themselves
        """
        self.window.updateText("File Name:\n")

        self.window.updateText(filenames[0] + '\n')
        self.window.targetname = filenames[0]


        return True #??? why need to return a bool??? ######


class TraceReaderPanel(wx.Panel): #panel for DnD

    def __init__(self, parent):
        """Constructor"""
        wx.Panel.__init__(self, parent=parent) #initialize
        self.targetname = ''
        self.tp = TracePlot(self)
        self.ScaleWindow = SetScaleFrame(self)
```

```
        self.createWidget()


    def createWidget(self):
        topsizer = wx.GridSizer(1, 10, 2, 2)
        self.Label_top = []
        self.Txt_top = []
        label_txt =
{0:'Average\nLength:',1:'Search\nLength:',2:'EWMA:',3:'Baseline\nLength:',4:'Stim\nStart:'}
        self.Txt_num = [10,30,3,100,100]#####
        Txt_txt = {0:str(self.Txt_num[0]),
                   1:str(self.Txt_num[1]),
                   2:str(self.Txt_num[2]),
                   3:str(self.Txt_num[3]),
                   4:str(self.Txt_num[4])}

        for i in range(5):
            self.Label_top.append(wx.StaticText(self, label=label_txt[i])) #add text
            self.Txt_top.append(wx.TextCtrl(self))
            topsizer.Add(self.Label_top[i], 0,wx.EXPAND)
            topsizer.Add(self.Txt_top[i], 0,wx.EXPAND)
            self.Txt_top[i].WriteText(Txt_txt[i])


        file_drop_target = MyFileDropTarget(self) #call function MyFileDropTarget
        self.fileTextCtrl = wx.TextCtrl(self,
                                        style=wx.TE_MULTILINE|wx.HSCROLL|wx.TE_READONLY)#add
text region for drop file
        self.fileTextCtrl.SetDropTarget(file_drop_target)#set this text for droping files
        self.fileTextCtrl.WriteText('Drag a .txt file to here. \n')
        self.fileTextCtrl.WriteText('File contents only numbers, 1st column is time or frame#. \n')

        leftsizer = wx.GridSizer(5, 1, 2, 2)
        self.Check_button_actions = []
        Check_button_actions_txt = {0:'Remove shifted cells',
                                    1:'backward subtraction',
                                    2:'save each Î”F/F0 traces after calculation',
                                    3:'save average trace after calculation'}
        check_button_actions_init = [False,True,True,False]
        for i in range(len(Check_button_actions_txt)):
            self.Check_button_actions.append(wx.CheckBox(self,
label=Check_button_actions_txt[i]))
            self.Check_button_actions[i].SetValue(check_button_actions_init[i])
            leftsizer.Add(self.Check_button_actions[i],0,wx.EXPAND)

        rightsizer = wx.GridSizer(5, 2, 2, 2)
```

```
self.Check_button_plots = []
check_txt = {0:'Raw Data',
            1:'Î”F/F0',
            2:'Average Î”F/F0',
            3:'Heatmap',}#4:'Active #'}
check_init = [True,True,True,True]

for i in range(len(check_txt)):
    self.Check_button_plots.append(wx.CheckBox(self, label=check_txt[i]))
    self.Check_button_plots[i].SetValue(check_init[i])

plotButton = wx.Button(self, label="quick plot")
plotButton.Bind(wx.EVT_BUTTON, self.pltButton_press)
heatmap_plotButton = wx.Button(self, label="quick plot")
heatmap_plotButton.Bind(wx.EVT_BUTTON, self.heatmap_plotButton_press)
rightsizer.AddMany([(self.Check_button_plots[0],0,wx.EXPAND),
                  (wx.StaticText(self, -1, ''),0,wx.EXPAND),
                  (self.Check_button_plots[1],0,wx.EXPAND),
                  (plotButton,0,wx.EXPAND),
                  (self.Check_button_plots[2],0,wx.EXPAND),
                  (wx.StaticText(self, -1, ''),0,wx.EXPAND),
                  (self.Check_button_plots[3],0,wx.EXPAND),
                  (heatmap_plotButton,0,wx.EXPAND)])

label_boxsizer = wx.BoxSizer(wx.HORIZONTAL)
heatmap_style_Label = wx.StaticText(self, label='Heatmap Style:')
label_boxsizer.Add(heatmap_style_Label,0,wx.RIGHT)
heatmap_link_Button = wx.Button(self, label="?", size = (20,25))
label_boxsizer.Add(heatmap_link_Button,0,wx.RIGHT)
heatmap_link_Button.Bind(wx.EVT_BUTTON, self.heatmap_link_Button_press)
self.heatmap_style_Txt = wx.TextCtrl(self)
self.heatmap_style_Txt.WriteText('rainbow')
rightsizer.AddMany([(label_boxsizer,0,wx.EXPAND),
                  (self.heatmap_style_Txt,0,wx.EXPAND)])


bottomsizer = wx.GridSizer(1, 2, 2, 2)
bottomsizer.Add(leftsizer,0,wx.EXPAND)
bottomsizer.Add(rightsizer,0,wx.EXPAND)


calButton = wx.Button(self, label="Î”F/F0 calculation")
calButton.Bind(wx.EVT_BUTTON, self.calButton_press)


sizer = wx.BoxSizer(wx.VERTICAL) #difine sizer
```

```
    sizer.Add(topsizer, 0, wx.ALL|wx.EXPAND, 5) #add first widget
    sizer.Add(self.fileTextCtrl, 1, wx.EXPAND|wx.ALL, 2) #add second widget

    sizer.Add(calButton, 0, wx.ALL|wx.EXPAND, 2) #add 4th widget
    sizer.Add(bottomsizer, 0, wx.ALL|wx.EXPAND, 2) #add 5th widget
    self.SetSizer(sizer) #set
def heatmap_link_Button_press(self,event):
    webbrowser.open('https://matplotlib.org/stable/gallery/color/colormap_reference.html')
def heatmap_plotButton_press(self,event):
    txtisnum = [self.Txt_top[i].GetLineText(0).isdigit() for i in range(4)]
    if np.sum(txtisnum) <3:
        self.fileTextCtrl.WriteText('***Please enter numbers*** \n')
    elif self.targetname == '':
        self.fileTextCtrl.WriteText('***Please drag a txt file to here*** \n')
    else:

        heatmap_style = self.heatmap_style_Txt.GetLineText(0)
        filedir, filebasename = os.path.split(self.targetname)
        traces = self.tp.dataRead(self.targetname)
        self.tp.heatmapPlot(filebasename,heatmap_style,traces)
        plt.show()

def pltButton_press(self,event):
    txtisnum = [self.Txt_top[i].GetLineText(0).isdigit() for i in range(4)]
    if np.sum(txtisnum) <3:
        self.fileTextCtrl.WriteText('***Please enter numbers*** \n')
    elif self.targetname == '':
        self.fileTextCtrl.WriteText('***Please drag a txt file to here*** \n')
    else:
        filedir, filebasename = os.path.split(self.targetname)
        traces = self.tp.dataRead(self.targetname)
        self.tp.dataPlot(filebasename,'quickplot',traces)
        plt.show()

        #---------------------------------------------------
def calButton_press(self,event):
    txtisnum = [self.Txt_top[i].GetLineText(0).isdigit() for i in range(4)]
    if np.sum(txtisnum) <3:
        self.fileTextCtrl.WriteText('***Please enter numbers*** \n')
    elif self.targetname == '':
        self.fileTextCtrl.WriteText('***Please drag a txt file to here*** \n')
    else:
        self.Txt_num = [int(self.Txt_top[i].GetLineText(0)) for i in range(5)]
        #threading plt to avoid block UI=====================
        self.fileTextCtrl.WriteText("Plot:\n")
        self.fileTextCtrl.WriteText(self.targetname + '\n')
```

```
            check_act_v = []
            check_plot_v = []
            for i in range(len(self.Check_button_actions)):
                check_act_v.append(self.Check_button_actions[i].GetValue())
            for i in range(len(self.Check_button_plots)):
                check_plot_v.append(self.Check_button_plots[i].GetValue())
            heatmap_style = self.heatmap_style_Txt.GetLineText(0)
            t = Thread
            t.__target = self.tp.dataPlotShow(self.targetname,
                                              self.Txt_num,
                                              check_act_v,
                                              check_plot_v,
                                              heatmap_style)
            try:
                t.start()
            except:
                self.fileTextCtrl.WriteText("close plot \n")
            #-----------------------------------------------------
    def SetInsertionPointEnd(self):
        self.fileTextCtrl.SetInsertionPointEnd()

    def updateText(self, text):
        self.fileTextCtrl.WriteText(text)
class SetScalePanel(wx.Panel):
    def __init__(self,parent):
        wx.Panel.__init__(self, parent=parent) #initialize
        self.createWidget()
    def createWidget(self):
        sizer = wx.GridSizer(1, 5, 2, 2)

        self.SetSizer(sizer) #set
class SetScaleFrame(wx.Frame):
    def __init__(self,parent):
        self.parent = parent
        wx.Frame.__init__(self, parent=None, title="Set scales", size=(500,500))
        panel = SetScalePanel(self)
        self.Hide()
#main frame for UI=====================================================
class TraceReaderFrame(wx.Frame):
    def __init__(self):
        """Constructor"""
        wx.Frame.__init__(self, parent=None, title="txt traces reader "+__version__,
size=(500,500))
        panel = TraceReaderPanel(self)
        self.Show()
```

```
#--------------------------------------------------------------------
if __name__ == "__main__":
    app = wx.App(False)
    frame = TraceReaderFrame()
    app.MainLoop()
```

Rotation behavior analysis

We used DeepLabCut™ (DLC) to analyze the recorded video. The process DLC was previously reported(citexxx). 200 frames of video were randomly selected for training. The snout, left ear, right ear and tail of mice were manually marked on these frames. After training, DLC can export the two-dimensional position of each mark in each frame based on transfer learning with deep neural networks. Then the position data was processed with a custom python script. In the script, we first picked up the frame with > 0.8 accuracy of position on snout and tail to identify the orientation of mice in each frame. When the accumulated angular change is larger than 360° clockwise, we regarded it as one clockwise rotation. Similarly, when the accumulated angular change is larger than 360°counterclockwise, we regarded it as one counterclockwise rotation. The time points of the ends of rotation were used for statistical analysis.

```
# Import assets
!pip install opencv-python
import numpy as np
import openpyxl
import pandas as pd
import math
from openpyxl import load_workbook

# Set the variables
basic_period = 300
stimulation_period = 600
time_period = 30
rest_period = 30
snout_index_on_sheet = 1
tail_index_on_sheet = 4

sheet_length = 9
date = "20211028"
angle_threshold = 360
likihood_threshold = 0.8
simulation_force = "50mT"
x1_cropping = 400
y1_cropping = 100
total_time = 900

snout_x_index= (snout_index_on_sheet - 1)*3 + 3
snout_y_index= (snout_index_on_sheet - 1)*3 + 4
snout_likihood_index= (snout_index_on_sheet - 1)*3 + 5
```

```
tail_x_index = (tail_index_on_sheet - 1)*3 + 3
tail_y_index = (tail_index_on_sheet - 1)*3 + 4
tail_likihood_index = (tail_index_on_sheet - 1)*3 + 5

sheets = []
mouse_title = []

# Load the sheets
for index in range(0, sheet_length):
    file_name = date + "-" + str(index+1)
    mouse_title.append("mice-"+str(index+1))
    read_file = pd.read_csv (file_name + ".csv")
    excel_file = read_file.to_excel (file_name + ".xlsx")
    excel_file = load_workbook(file_name + ".xlsx")
    sheet = excel_file.active
    sheets.append(sheet)

# Translate two-dimensional position into vector, angle and velocity
def Data_Translate(sheet):
        row_count = sheet.max_row
        all_data = []
        frame = []
        body_vector = []
        body_position = []
        angle = []
        vector = []
        velocity = []
        distance = []
        counter_clockwise_rotation_accumulation = []
        clockwise_rotation_accumulation = []
        current_frame_range = 1
        current_angle_accumulatation = 0
        counter_clockwise_rotation = 0
        clockwise_rotation = 0
        basic_counter_clockwise_rotation = 0
        basic_clockwise_rotation = 0
        stimulation_counter_clockwise_rotation = 0
        stimulation_clockwise_rotation = 0
        current_velocity_accumulation = 1
        current_velocity_range = 1
        distance_accumulation = 0

        #Pick the frame which its accuracy is greater than 0.8
    for rownum in range(4, row_count):
                if float(sheet.cell(rownum, snout_likihood_index).value) >= likihood_threshold and
        float(sheet.cell(rownum, tail_likihood_index).value) >= likihood_threshold:
```

```
            frame.append(float(sheet.cell(rownum, 2).value)
            body_vector.append([float(sheet.cell(rownum, tail_x_index).value)-
        float(sheet.cell(rownum, snout_x_index).value), float(sheet.cell(rownum,
        tail_y_index).value)-float(sheet.cell(rownum, snout_y_index).value)])
            body_position.append([(int((float(sheet.cell(rownum, tail_x_index).value)+
        float(sheet.cell(rownum, snout_x_index).value))/2)), (int((float(sheet.cell(rownum,
        tail_y_index).value)+ float(sheet.cell(rownum, snout_y_index).value))/2))])

    #Translate two-dimensional position into vector
for index in range(1, len(body_vector)):
        unit_vector_1 = body_vector[index-1] / np.linalg.norm(body_vector[index-1])
        unit_vector_2 = body_vector[index] / np.linalg.norm(body_vector[index])
        the_norm = np.linalg.norm(body_vector[index-
1])*np.linalg.norm(body_vector[index])
        rho = np.rad2deg(np.arcsin(np.cross(body_vector[index-1],
body_vector[index])/the_norm))
        theta = np.rad2deg(np.arccos(np.dot(body_vector[index-
1],body_vector[index])/the_norm))
        if rho < 0:
            angle.append(-theta)
            vector.append(-math.sqrt(math.pow(body_position[index][0]-
        body_position[index-1][0],2)+math.pow(body_position[index][1]-
        body_position[index-1][1],2))/(frame[index]-frame[index-1]))
        else:
            angle.append(theta)
            vector.append(math.sqrt(math.pow(body_position[index][0]-
        body_position[index-1][0],2)+math.pow(body_position[index][1]-
        body_position[index-1][1],2))/(frame[index]-frame[index-1]))

    #Calculate the angular change and accumulate the change
for index in range(0, len(angle)):
        if frame[index+1] >= time_period*frame_per_second*current_frame_range:
            counter_clockwise_rotation_accumulation.append(counter_clockwise_rotation)
            clockwise_rotation_accumulation.append(clockwise_rotation)
            counter_clockwise_rotation = 0
            clockwise_rotation = 0
            current_frame_range += 1
            current_angle_accumulatation += angle[index]
        if current_angle_accumulatation >= angle_threshold:
            counter_clockwise_rotation += 1
            current_angle_accumulatation = 0
elif current_angle_accumulatation <= angle_threshold*(-1):
            clockwise_rotation += 1
            current_angle_accumulatation = 0

    #Calculate the number of rotation during baseline and stimulation
```

```
        for index in range(0, len(counter_clockwise_rotation_accumulation)):
            if index <= basic_period/time_period:
                basic_counter_clockwise_rotation +=
            counter_clockwise_rotation_accumulation[index]
                basic_clockwise_rotation += clockwise_rotation_accumulation[index]
          elif index > basic_period/time_period and index <= basic_period/time_period +
stimulation_period/time_period:
                stimulation_counter_clockwise_rotation +=
            counter_clockwise_rotation_accumulation[index]
                stimulation_clockwise_rotation += clockwise_rotation_accumulation[index]

          # Translate two-dimensional position into velocity
    for index in range(1, len(body_position)):
            if frame[index] >= basic_period*frame_per_second*current_velocity_range or index
        == len(body_position)-1:
                distance_accumulation = distance_accumulation * 0.026
                distance.append(distance_accumulation)
                velocity.append(distance_accumulation/basic_period)
                distance_accumulation = 0
                current_velocity_range  += 1
                distance_accumulation += (math.sqrt(math.pow(body_position[index][0]-
            body_position[index-1][0],2)+math.pow(body_position[index][1]-
            body_position[index-1][1],2)))

        frame_data = np.array(frame)
        angle_data = np.array(angle)
        counter_clockwise_rotation_data = np.array(counter_clockwise_rotation_accumulation)
        clockwise_rotation_data = np.array(clockwise_rotation_accumulation)
        velocity_data = np.array(velocity)
        distance_data = np.array(distance)
        body_position_data = np.array(body_position)

    all_data.append(frame)
    all_data.append(angle)
    all_data.append(counter_clockwise_rotation_accumulation)
    all_data.append(clockwise_rotation_accumulation)
    all_data.append(velocity)
    all_data.append(distance)
    all_data.append(basic_counter_clockwise_rotation)
    all_data.append(basic_clockwise_rotation)
    all_data.append(stimulation_counter_clockwise_rotation)
    all_data.append(stimulation_clockwise_rotation)
    all_data.append(body_position)
    all_data_data = np.array(all_data, dtype = object)
    return frame_data, angle_data, counter_clockwise_rotation_data, clockwise_rotation_data,
velocity_data, distance_data, basic_counter_clockwise_rotation, basic_clockwise_rotation,
```

```
stimulation_counter_clockwise_rotation, stimulation_clockwise_rotation, body_position_data,
all_data_data


# Save data
frame_all_sheet = []
basic_counter_clockwise_rotation_all_sheet = []
basic_clockwise_rotation_all_sheet = []
stimulation_counter_clockwise_rotation_all_sheet = []
stimulation_clockwise_rotation_all_sheet  = []
velocity_all_sheet = []
distance_all_sheet = []
rotation_all_sheet = []
body_position_all_sheet = []
sheet_data = []
all_data_all_sheet = []

mouse_title_rotation = []
for index in range(0, len(sheets)):
    all_data_all_sheet.append(Data_Translate(sheets[index])[11])
    frame_all_sheet.append(all_data_all_sheet[index][0])
    rotation_all_sheet.append(all_data_all_sheet[index][2])
    rotation_all_sheet.append(all_data_all_sheet[index][3])
    velocity_all_sheet.append(all_data_all_sheet[index][4])
    distance_all_sheet.append(all_data_all_sheet[index][5])
        basic_counter_clockwise_rotation_all_sheet.append(all_data_all_sheet[index][6])
    basic_clockwise_rotation_all_sheet.append(all_data_all_sheet[index][7])
        stimulation_counter_clockwise_rotation_all_sheet.append(all_data_all_sheet[index][8])
        stimulation_clockwise_rotation_all_sheet.append(all_data_all_sheet[index][9])
        body_position_all_sheet.append(all_data_all_sheet[index][10])

for index in range(0, len(mouse_title)):
    mouse_title_rotation.append("mice-"+str(math.floor(index/2)+1)+"- counterclockwise")
    mouse_title_rotation.append("mice -"+str(math.floor(index/2)+1)+"- clockwise")
data = {
    "basic_counter_clockwise":basic_counter_clockwise_rotation_all_sheet,
    "basic_clockwise":basic_clockwise_rotation_all_sheet,
        "stimulation_counter_clockwise":stimulation_counter_clockwise_rotation_all_sheet,
    "stimulation_clockwise":stimulation_clockwise_rotation_all_sheet
        }

data1 = pd.DataFrame(data)
data2 = pd.DataFrame(rotation_all_sheet)
data3 = pd.DataFrame(velocity_all_sheet)
data4 = pd.DataFrame(distance_all_sheet)
df1 = data1.rename(lambda x: (x+1)/2)
```

```
df2 = data2.rename(lambda x: (x+1)/2)
df3 = data3.rename(lambda x: (x+1)/2)
df4 = data4.rename(lambda x: (x+1)/2)
df1 = df1.transpose()
df1.columns = mouse_title
df2 = df2.transpose()
df2.columns = mouse_title_rotation
df3 = df3.transpose()
df3.columns = mouse_title
df4 = df4.transpose()
df4.columns = mouse_title
with pd.ExcelWriter(str(date) + "-" +  str(likihood_threshold) + "-" + simulation_force +'-output-distance.xlsx') as writer:
    df1.to_excel(writer, sheet_name='rotation-period')
    df2.to_excel(writer, sheet_name='rotation-every-frame')
    df3.to_excel(writer, sheet_name='velocity-period')
    df4.to_excel(writer, sheet_name='distance-period')
```
