# Supplementary material for: Wireless neuromodulation in vitro and in vivo by intrinsic TRPC-mediated magnetomechanical stimulation
Source: Commun Biol. 2022 Nov 2;5:1166. doi: 10.1038/s42003-022-04124-y (PMC9630493; doi:10.1038/s42003-022-04124-y)
Supplement: Supplementary file 3 — Description of Additional Supplementary Files [file 42003_2022_4124_MOESM3_ESM.pdf]

Description of Additional Supplementary Files

File Name: Supplementary Data 1

Description: Source data underlying figures used in the study are provided in Supplementary Data 1.
