# Supplementary material for: Wireless neuromodulation in vitro and in vivo by intrinsic TRPC-mediated magnetomechanical stimulation
Source: Commun Biol. 2022 Nov 2;5:1166. doi: 10.1038/s42003-022-04124-y (PMC9630493; doi:10.1038/s42003-022-04124-y)
Supplement: Supplementary file 5 — Reporting Summary [file 42003_2022_4124_MOESM5_ESM.pdf]

nature portfolio

Corresponding author(s): Po-Han Chiang
Last updated by author(s): Sep 21, 2022

Reporting Summary

Nature Portfolio wishes to improve the reproducibility of the work that we publish. This form provides structure for consistency and transparency in reporting. For further information on Nature Portfolio policies, see our Editorial Policies and the Editorial Policy Checklist.

Statistics

For all statistical analyses, confirm that the following items are present in the figure legend, table legend, main text, or Methods section.

n/a	Confirmed	
☐	☒	The exact sample size (*n*) for each experimental group/condition, given as a discrete number and unit of measurement
☐	☒	A statement on whether measurements were taken from distinct samples or whether the same sample was measured repeatedly
☐	☒	The statistical test(s) used AND whether they are one- or two-sided<br>*Only common tests should be described solely by name; describe more complex techniques in the Methods section.*
☐	☒	A description of all covariates tested
☐	☒	A description of any assumptions or corrections, such as tests of normality and adjustment for multiple comparisons
☐	☒	A full description of the statistical parameters including central tendency (e.g. means) or other basic estimates (e.g. regression coefficient) AND variation (e.g. standard deviation) or associated estimates of uncertainty (e.g. confidence intervals)
☐	☒	For null hypothesis testing, the test statistic (e.g. *F*, *t*, *r*) with confidence intervals, effect sizes, degrees of freedom and *P* value noted<br>*Give P values as exact values whenever suitable.*
☒	☐	For Bayesian analysis, information on the choice of priors and Markov chain Monte Carlo settings
☐	☒	For hierarchical and complex designs, identification of the appropriate level for tests and full reporting of outcomes
☒	☐	Estimates of effect sizes (e.g. Cohen's *d*, Pearson's *r*), indicating how they were calculated

Our web collection on statistics for biologists contains articles on many of the points above.

Software and code

Policy information about availability of computer code

Data collection	HC image(version 4.6.1.3)<br>FEMM(version 4.2)<br>Arduino(version 1.8.13)<br>Arduino script for controlling in vivo coil system is described in supplementary information
Data analysis	JASP (version 0.14.1.0)<br>Image j(version 1.53k)<br>Python(version 3.7)<br>Python script for fluorescence intensity analysis is described in supplementary information

For manuscripts utilizing custom algorithms or software that are central to the research but not yet described in published literature, software must be made available to editors and reviewers. We strongly encourage code deposition in a community repository (e.g. GitHub). See the Nature Portfolio guidelines for submitting code & software for further information.

Data

Policy information about availability of data

All manuscripts must include a data availability statement. This statement should provide the following information, where applicable:

- Accession codes, unique identifiers, or web links for publicly available datasets
- A description of any restrictions on data availability
- For clinical datasets or third party data, please ensure that the statement adheres to our policy

All software we used can be download from website below(websites are available when writing).
- HC image(https://hcimage.com/download/)
- FEMM(https://www.femm.info/wiki/Download)
- Arduino(https://www.arduino.cc/en/software)
- JASP (https://jasp-stats.org/)
- Image j(https://imagej.nih.gov/ij/download.html)

Arduino code were written inside of supplementary.
No restrictions on data availability

Human research participants

Policy information about studies involving human research participants and Sex and Gender in Research.

Reporting on sex and gender	N/A
Population characteristics	N/A
Recruitment	N/A
Ethics oversight	N/A

Note that full information on the approval of the study protocol must also be provided in the manuscript.

Field-specific reporting

Please select the one below that is the best fit for your research. If you are not sure, read the appropriate sections before making your selection.

☒ Life sciences ☐ Behavioural & social sciences ☐ Ecological, evolutionary & environmental sciences

For a reference copy of the document with all sections, see nature.com/documents/nr-reporting-summary-flat.pdf

Life sciences study design

All studies must disclose on these points even when the disclosure is negative.

Sample size	For in vitro test, we chose sample size around 5 to 10 due to limited cultured cell but variety of experimental groups and control group. As for in vivo test, the sample size is 10. According to the principle of 3R's for animal test in NYCU IACUC, reduce as much as possible animal used. Hence we chose the sample size equal to 10 to provide enough data and also following the 3R's rules.
Data exclusions	This study did not exclude data.
Replication	All results in the study can be repeated and the repeated outcomes corresponded to the original data.
Randomization	Cultured cells were from pups of pregnant Sprague-Dawley rats less than 3 day. In vivo test of C57BL/6 mice were older than 8 weeks. In the study, no selection of which cultured cell or mice to do the experiments.
Blinding	In the study, blinding is not possible and relevant. Researchers added nanodiscs on cultured cells, performed injection surgeries and make brain slices. Knowing the groups in experiments did not affect the results.

Reporting for specific materials, systems and methods

We require information from authors about some types of materials, experimental systems and methods used in many studies. Here, indicate whether each material, system or method listed is relevant to your study. If you are not sure if a list item applies to your research, read the appropriate section before selecting a response.

Materials & experimental systems

n/a	Involved in the study
☐	☒ Antibodies
☒	☐ Eukaryotic cell lines
☒	☐ Palaeontology and archaeology
☐	☒ Animals and other organisms
☒	☐ Clinical data
☒	☐ Dual use research of concern

Methods

n/a	Involved in the study
☒	☐ ChIP-seq
☒	☐ Flow cytometry
☒	☐ MRI-based neuroimaging

Antibodies

Antibodies used	Primary antibody: anti-c-Fos antibody, anti-Neun antibody, anti-TRPC1 antibody, anti-TRPC5 antibody, anti-TRPC6 antibody<br>Secondary antibody: goat anti-rabbit Alexa Fluor 488, goat anti-rabbit Alexa Fluor 594
Validation	Rabbit anti-c-Fos monoclonal antibody (9F6#2250, Cell signaling)<br>Mouse anti-NeuN antibody (clone A60, #MAB377, MERCK)<br>Anti-rabbit NeuN (MABN140, Sigma-Aldrich)<br>Anti-rabbit TRPC1 (SI-T8276, Sigma-Aldrich)<br>Anti-mouse TRPC5 (N67/15, NeuroMab)<br>Anti-rabbit TRPC6 (AB5574, MERCK)<br>Goat anti-rabbit Alexa Fluor 488 (ab150113, Abcam)<br>Goat anti-mouse Alexa Fluor 594 (ab150116, Abcam)

Animals and other research organisms

Policy information about studies involving animals; ARRIVE guidelines recommended for reporting animal research, and Sex and Gender in Research

Laboratory animals	All pregnant Sprague-Dawley rats and C57BL/6 male mice were from LASCO, and animals were maintained under a 12 h light-dark cycle at NYCU Laboratory Animal Center before experiment.
Wild animals	The study did not involve wild animals.
Reporting on sex	Cultured cells from neutral gender pups of Sprague-Dawley rats were used for in vitro test.<br>C57BL/6 male mice were used for in vivo test.
Field-collected samples	The study did not involve sample collected from the field.
Ethics oversight	All the animal experiments were approved by NYCU IACUC, in accordance with the Guide for the Care and Use of Laboratory Animals of NYCU.

Note that full information on the approval of the study protocol must also be provided in the manuscript.
